# The Impact of B-Cell Perturbations on Pneumococcal Conjugate Vaccine Response in HIV-Infected Adults

**DOI:** 10.1371/journal.pone.0042307

**Published:** 2012-07-30

**Authors:** Thomas G. Johannesson, Ole S. Søgaard, Martin Tolstrup, Mikkel S. Petersen, Jens M. Bernth-Jensen, Lars Østergaard, Christian Erikstrup

**Affiliations:** 1 Department of Infectious Diseases, Aarhus University Hospital, Aarhus, Denmark; 2 Department of Clinical Immunology, Aarhus University Hospital, Aarhus, Denmark; Health Protection Agency, United Kingdom

## Abstract

Untreated HIV infection results in severe perturbations of the B-cell population and hyporesponsiveness to vaccination. We studied associations between circulating B-cell subsets and antibody response to pneumococcal conjugate vaccine in treated and untreated HIV patients.

Ninety-five HIV-infected adults were grouped according to antiretroviral therapy (ART) and CD4+ cell count as follows: 20 ART-naïve (no prior ART), 62 ART-responders (received ART, and CD4 count >500 cells/µl), and 13 impaired responders (received ART for more than 3 years, and CD4 count <500 cells/µl). All subjects were immunized twice with double-dose 7-valent pneumococcal conjugate vaccine with or without 1 mg CPG 7909 (toll-like receptor 9 agonist) at baseline and after three months. Pre-vaccination B-cell subpopulations were assessed by flow cytometry. Serum IgG concentrations for vaccine serotypes were quantified by ELISA at baseline and 3, 4, and 9 months post-vaccination. ART responders had more isotype-switched memory B cells and more marginal-zone (MZ)-like B cells compared with impaired responders. Furthermore, ART-naïve patients had higher concentration of transitional B cells and plasmablasts compared with B cells of other patient groups. The concentration of MZ-like, isotype switched memory cells and plasmablasts correlated positively with post-vaccination IgG concentration at 3, 4, and 9 months. Low concentrations of isotype-switched memory B cells was the strongest independent predictor of poor pneumococcal conjugate vaccine responsiveness, emphasizing that B-cell subset disturbances are associated with poor vaccine response among HIV-infected patients

## Introduction

Persons with HIV infection have a six- to eight-fold increased risk of pneumonia [1], [2] and up to a 40-fold higher risk of invasive pneumococcal disease compared with healthy individuals [3]. The U. S. National Institutes of Health recommend vaccination of HIV patients with the 23-valent polysaccharide vaccine (PPV-23) [4]. However, a higher risk of pneumococcal infection persists, even among vaccinated individuals [5], [6]. Although use of antiretroviral therapy (ART) improves the response to pneumococcal vaccination [7], [8], post-vaccination antibody levels are lower among ART-treated individuals compared with HIV-uninfected persons [9], [10].

The main protective mechanism against invasive pneumococcal disease is opsonizing, serotype-specific anticapsular immunoglobulins (Ig), although other factors, such as non-capsular antibodies and interleukin (IL)-17-producing CD4 T cells, may also play a role [11].

HIV infection affects humoral immunity both indirectly, through reduced T-cell help, and directly, through changes in the B-cell compartment [12]. These changes include polyclonal activation of the B-cell pool, dysregulation of isotype switching, structural damage to secondary lymphoid organs, and perturbations in the proportions and absolute numbers of circulating B-cell subsets [12], [13] leading to reduced responsiveness to immunization [13], [14].

IgM^+^ memory B cells are central in immune responses to T-cell-independent antigens, such as pneumococcal capsular polysaccharides [15]. The IgM memory B cell type was originally regarded as a memory subset because these B cells possess somatically hypermutated IgM receptors [16]. However, more recent findings suggest that these cells are a recirculating counterpart of splenic marginal zone (MZ) B cells and possess a pre-diversified immune repertoire capable of responding rapidly to encapsulated bacteria [15], [17].

Initiation of suppressive ART reduces polyclonal B-cell activation [12], [18] and normalizes the numbers of both circulating transitional and naïve B cell [12], [13], [19], [20]. However, neither the amount of circulating isotype-switched memory B cells nor their functions are restored by ART [14], [21] which may affect antibody-mediated immunity, even in well-treated HIV patients [9], [10].

The seven-valent pneumococcal conjugate vaccine (PCV-7) protects against invasive pneumococcal disease in HIV infected adults [22]; furthermore, addition of toll-like receptor 9 (TLR9) agonist (CPG 7909) as an adjuvant to PCV-7 increases the odds of HIV-infected patients achieving a high vaccine-specific IgG antibody response [23]. The aim of this study was to delineate B-cell subsets in treated and untreated HIV patients and to determine the association of these subsets with antibody responses to PCV-7 administered with or without a TLR9 agonist.

## Methods

### Study design and ethics statement

This observational study was a sub-study to an investigator-initiated phase Ib/IIa, randomized, double-blind, placebo-controlled trial, in which HIV-infected adults were immunized with pneumococcal vaccines with or without CPG 7909 [23]. The Danish Medicines Agency, the Regional Ethics Committee, and the Danish Data Protection Agency approved the study protocols. Studies were registered at www.clinicaltrials.gov (NCT00562939). All participants provided written informed consent.

### Setting and participants

The study was conducted at the Department of Infectious Diseases, Aarhus University Hospital, Skejby, Denmark. The study population included 95 HIV-seropositive volunteers aged 18 years or older. Three distinct patient groups were identified within the cohort. Patients never treated with antiretroviral drugs were categorized as “ART-naïve” (n = 20). Patients who received ART for a minimum of three years without reaching a CD4 T cell count above 500 cells/µl were classified as immunologically “impaired responders” (n = 13). Finally, patients on ART who achieved a CD4 T cell count above 500 cells/µl were defined immunologically as “ART-responders” (n = 62).

 To obtain a well-defined and representative population, we excluded individuals who had received PPV-23 immunization within the last five years, were on antiretroviral therapy for less than six months, were on antiretroviral therapy with HIV RNA above 50 copies/ml, had a CD4 T cell count below 200 cells/µl, or were unavailable for first follow-up visit after first immunization.

### Immunization and sample collection

**Figure 1:**
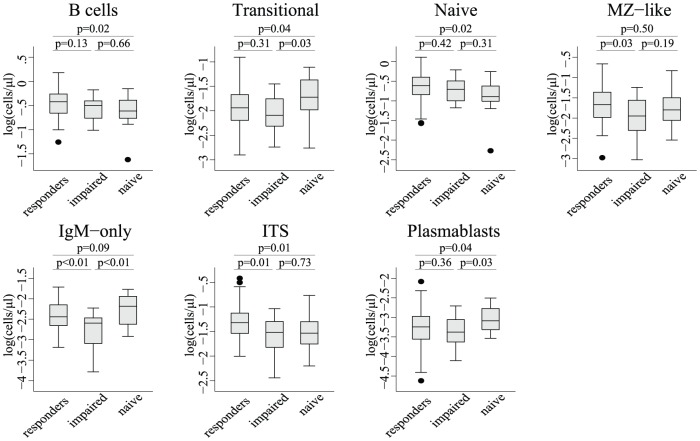
Pre-vaccination difference in B cell subpopulations between patient groups: the impact of successful ART. Box plots showing the absolute cell count of B-cell populations in the three patient groups (naïve; never treated with antiretroviral therapy (ART), impaired; ART for a minimum of 3 years, and still not achieving a CD4 T cell count above 500 cells/ml, responders; ART and a CD4 T cell count above 500 cells/ml). The vertical axis displays the logarithmic transformed cell count in cells/ul, and the horizontal line in the box indicates the median value of the group; whiskers illustrate the interquartile ranges. Populations are compared using a Student's T-test and p-values express the level of significant difference between groups. One outlier has been removed from the plasmablasts. This ART-naïve patient had a value of −4.20 log(cells/µl).

All participants were immunized with twice the standard dose of PCV-7 (Prevnar, Wyeth) at baseline and three months later. Participants were seen 4 and 9 months after first immunization for blood sampling and safety follow-up (Figure 1). One group received 1.0 mg CPG7909 (formulated in 100 µl phosphate buffered saline (PBS)) added to each of their two vaccine doses, whereas the other group received placebo (PBS) added to the vaccine doses. Before vaccination and at the 3, 4, and 9-month follow-up visits, we collected serum for antibody measurements and routine laboratory tests including HIV RNA level (Roche Amplicor; F. Hoffmann-La Roche, Basel, Switzerland) and CD4 T-cell counts (FACSCalibur or FACSCanto; Becton-Dickinson, San Jose, CA). Peripheral blood mononuclear cells (PBMCs) were isolated from sodium citrate-stabilized whole blood in 8-ml cell preparation tubes (CPT Vacutainer, BD Biosciences, Franklin Lakes, NJ) according to the manufacturer's instructions. PBMCs were resuspended in RPMI 1640 medium (Sigma-Aldrich, Seelze, Germany) with 10% dimethyl sulfoxide and 20% heat-inactivated fetal calf serum before cryopreservation at −170°C.

### Assessment of immunogenicity

Serotype-specific IgG serum concentrations for PCV-7 serotypes (4, 6B, 9V, 14, 18C, 19F, and 23F) were analyzed in one batch by ELISA [24]. Serum samples were preadsorbed with optimal concentrations of pneumococcal cell wall and 22F-capsular polysaccharides [25]. We used the WHO recommended ELISA for quantitation of Streptococcus pneumoniae serotype specific IgG which has two adsorption steps: the CWPS (C-Ps, Teichoic acid) and the 22F pneumococcal capsule. For further information please refer to http://www.vaccine.uab.edu/ELISAProtocol(89SF).pdf

Opsonophagocytic activity (OPA) of anticapsular antibodies (serotypes 6B, 14, 19F, and 23F) was measured using a flow cytometric opsonophagocytic assay giving indirect information on the antibodies' ability to opsonize and facilitate killing of invading pneumococci [26] (Flow applications Inc., Illinois, USA). Serum samples were stored at −80°C before shipment for analysis.

### Cell staining and flow analysis

Multicolour flow cytometry was performed in batches of eight to ten patient samples per day. The cryopreserved PBMCs were rapidly thawed and transferred into 30 ml cold (+5°C) buffered saline solution (BSS). After a double wash, cells were resuspended in FACS tubes (Becton Dickinson) with a staining buffer containing bovine serum albumin and incubated for 20 minutes with the following antibodies (BD Biosciences): α-CD19 (clone 4G7) and α-CD20 (clone L27) conjugated peridinin chlorophyll protein complex (PerCP), α-IgD (clone IA6-2) conjugated phycoerythrin (PE), α-IgM (clone G20-127) conjugated fluorescein isothiocyanate (FITC), α-CD38 (clone HB7) conjugated PE-cyanine 7 (Cy7), α-CD27 (clone L128) conjugated allophycocyanin (APC), and αCD3 (clone SK7) conjugated APC-H7. Samples were subsequently washed with 2 ml FACS flow buffer (BD Biosciences) and analyzed on a FACSCanto flow cytometer (BD Biosciences). Samples were compensated for spectral overlap with BD CompBeads (BD Biosciences) and relevant antibodies.

Data were analyzed with FlowJo v.9.2 (Tree Star, Ashland, OR) software (Figure S1). A forward-scatter height versus forward-scatter area and subsequent forward-scatter versus side-scatter plot was used to define events that represent lymphocytes. B cells were defined as CD19/CD20 positive and CD3 negative and subcategorized using quadrant gates based on expression of IgD and IgM: IgD-only (IgD^+^, IgM^−^), double-positive (IgD^+^, IgM^+^), IgM-only (IgD^−^, IgM^+^), and double-negative/isotype switched (IgD^−^, IgM^−^) cells. Each quadrant was subsequently plotted on a CD38 versus CD27 plot in which new quadrant gates were applied.

The B-cell subsets were characterized as follows [27], [28]: transitional B cells were defined as IgD^+^, IgM^+/−^, CD27^−^, and CD38^+^; naïve B cells were IgD^+^, IgM^+/−^, CD27^−^, and CD38^−^; MZ-like B cells were IgD^+^, IgM^+^, CD27^+^, and CD38^−^. Memory B populations were CD27^+^ and CD38^−^, whereas plasmablasts were CD27^+^ and CD38^+^. Based on their surface Ig expression, the populations were divided into isotype-switched (IgD^−^, IgM^−^) and IgM-only (IgD^−^, IgM^+^). Results were expressed as B-cell concentrations, calculated as the fraction of the leukocyte count measured on the same day by routine lab analysis, or as subset percentage of the total B-cell fraction. The cell staining, data capture, and subsequent flow analysis were performed in a blinded fashion.

### Statistical analysis

We calculated the vaccine antibody responses as the relative vaccine-specific IgG increase from pre-vaccination baseline to 3, 4, and 9 months, thus providing a correlate for total vaccine response adjusted for the patient's IgG concentration of the seven vaccine serotypes at the time of enrolment. The mean values of the seven PCV-7 serotype-specific IgG concentrations at 3, 4, and 9 months were calculated and each was divided by the mean baseline PCV-7 serotype-specific IgG concentration [8], [23].

Similarly, the vaccine-specific OPA response for serotypes 6B, 14, 19F, and 23F was calculated as the mean value of the four measured serotypes at 3, 4 and 9 months respectively, divided by the mean baseline OPA titer.

The number of B cells and B-cell subpopulations were compared between the three patient groups by Student's T-test.

Correlations between the relative increase in PCV-7-specific IgG concentration or the OPA titer at the 3, 4, and 9-month follow-up visits and the pre-vaccination baseline B cell variables were evaluated by univariate linear regression analysis.

This was supplemented with multivariable regression analyses with adjustments for patient group (“ART-naïve”, “impaired responders”, “ART-responders”), CPG7909 adjuvant (yes/no), and current smoker (yes/no). Initially, adjustment for age was included in all analyses, but because this did not alter any results, age was excluded in the final analyses.

All variables were checked for normal distribution using quantile normalization. Logarithmic transformation was performed when appropriate to obtain normality.

Pre-vaccination baseline characteristics are presented as median values with interquartile ranges (IQR) for the continuous variables. Categorical variables are presented as counts and percentages.

P-values below 0.05 were considered significant. Statistical analyses were performed using STATA v. 11.1 (StataCorp, College Station, TX).

## Results

### Study population

The 95 patients categorized as ART-naïve, ART impaired responders or ART responders are characterized in Table 1. Forty-seven patients (49.5%) received CPG7909 adjuvant with their pneumococcal vaccination. Four patients were lost to follow-up.

**Table 1:** Baseline characteristics of the study population at the time of inclusion.

		HIV cohort	ART-responders	Impaired responders	ART-naive
		n = 95	n = 62	n = 13	n = 20
Age, median (IQR)		49.1 (42.6–59.2)	48.9 (41.8–59.2)	49.6 (43.6–61.8)	48.2 (40.6–55.3)
Sex, no (%)					
	Male	80 (84.2)	55 (88.7)	9 (69.2)	16 (80.0)
	Female	15 (15.8)	7 (11.3)	4 (30.8)	4 (20.0)
Race, no (%)					
	Caucasian	89 (93.7)	59 (95.2)	11 (84.6)	19 (95.0)
	Non-caucasian	6 (6.3)	3 (4.8)	2 (15.4)	1 (5.0)
Current smoker, no (%)		34 (35.8)	22 (35.5)	3 (23.1)	9 (45)
BMI, median (IQR)		23.7 (22.0–25.8)	23.2 (21.6–24.9)	24.3 (23.3–25.9)	24.6 (22.6–26.5)
CD4 count median (IQR)		632.4 (458.4–833.8)	727.2 (564.3–871.7)	408.3 (319.7–442.1)	496.8 (372.6–812.2)
ART duration, median years (IQR)		8.30 (3.01–10.73)	7.21 (2.67–10.73)	8.55 (6.93–9.82)	NA
Nadir CD4 median (IQR)		220.0 (100.0–307.0)	215.5 (100.0–257.0)	50.0 (20.0–136.0)	401.0 (313.0–659.0)
HIV RNA median log10 (IQR)			<1.6 (2.2)	<1.6 (1.9)	4.3 (3.7–4.7)
Randomized to CPG7909 adjuvant, no (%)		47 (49.5)	28 (45.2)	9 (69.2)	10 (50.0)
History of invasive *Streptococcus Pneumoniae*, no		0	0	0	0

ART; antiretroviral therapy, CPG 7909; toll-like receptor 9 agonist, IQR; Interquartile range, BMI; body mass index, PPV-23; 23-valent pneumococcal polysaccharide vaccine.

### B cell counts were lower among ART-naïve patients than treated patients

ART-naïve participants had lower B cell concentrations compared with ART responders (p = 0.02, Figure 1). Furthermore, the ART-naive patients had lower numbers of naive B cells, compared with the ART responders (p = 0.02, Figure 1).

### Memory B cell subsets were diminished among impaired responders compared to responders

Impaired responders had lower MZ-like B-cell counts compared with ART responders (p = 0.03, Figure 1). This was not observed for the MZ-like percentage of B cells (data not shown). No differences were found in the MZ-like B-cell count or the MZ-like percentage of B cells between ART-naïve patients and the two groups of treated patients.

The concentration of IgM-only memory cells was higher among ART responders and ART-naïve patients compared with impaired responders (p<0.01, Figure 1).

ART responders had higher isotype-switched memory-cell counts compared with impaired responders and ART-naïve patients (p = 0.01 in both analyses, Figure 1), whereas there was no difference between ART-naïve patients and impaired responders. The percentage of isotype-switched memory B cells did not differ between the groups (data not shown).

### Increased B cell turnover in ART-naïve compared with treated patients

ART-naïve patients had higher concentrations (Figure 1) and percentages (data not shown) of transitional B cells and isotype-switched plasmablasts compared with the treated groups (p<0.05 in all analyses). There was no difference between ART responders and impaired responders.

### Total B cells demonstrated no correlation with vaccine-specific IgG response

In unadjusted linear regression analyses, the absolute B cell count did not predict the vaccine response as measured by the serotype-specific IgG concentration at 3, 4, and 9 months after PCV-7 vaccination (p>0.05 in all analyses, data not shown). Similarly, regression analyses adjusted for patient group, CpG adjuvant, and current smoking status showed no effect of total B-cell concentration on IgG vaccine response (p>0.05 in all analyses, Table 2). Furthermore, there was no association between naïve B cells with the plasma IgG concentration in unadjusted or adjusted regression analyses (p>0.05 in all analyses).

**Table 2:** Adjusted regression analysis: Baseline B-cell subpopulations as predictors for IgG antibody titers.

	3 months, n = 95	4 months, n = 92	9 months, n = 91
B cell subset	RC (p)	RC (p)	RC (p)
Total B cells	0.17 (0.19)	0.16 (0.23)	0.20 (0.13)
Transitional	0.10 (0.28)	0.09 (0.32)	0.11 (0.25)
Naive	0.06 (0.60)	0.05 (0.64)	0.08 (0.45)
MZ-like	**0.20 (0.01)**	**0.20 (0.01)**	**0.21 (0.01)**
IgM-only memory	0.19 (0.07)	0.17 (0.08)	0.15 (0.13)
ITS memory	**0.38 (<0.01)**	**0.32 (<0.01)**	**0.35 (<0.01)**
ITS Plasmablasts	**0.20 (0.02)**	**0.20 (0.02)**	**0.17 (0.03)**

IgG antibody concentration is calculated as the relative vaccine-specific IgG increase of the 7 *Streptococcus pneumoniae* polysaccharide serotypes contained in the PCV-7 vaccine from pre-vaccination baseline to 3, 4 and 9 months, measured by ELISA.

B-cell counts are calculated as the routine lab lymphocyte count multiplied with the B-cell percentage of lymphocytes measured by flow cytometry.

Adjusted for patient group (‘ART-naïve’, ‘impaired responders’, ‘ART-responders’), CPG7909 adjuvant (yes/no) and current smoker (yes/no).

RC; Regression Coefficient (increase in log(µg IgG/ml) with 10-fold increase in cell counts/fractions), p; p-value, n; number of participants.

Transitional; IgD+, IgM+, CD27−, CD38+ B cells, Naive; IgD+, IgM+/−, CD27−, CD38− B cells, MZ-like; marginal zone-like B cells (IgD+, IgM+, CD27+, CD38−), IgM-only memory; IgD−, IgM+, CD27+, CD38− B cells, ITS memory; isotype switched memory B cells (IgD−, IgM−, CD27+, CD38−), ITS plasmablasts; isotype switched plasmablasts (IgD−, IgM−, CD27+, CD38+).

### Isotype switched memory B cells strongly predicted vaccination responses

**Figure 2:**
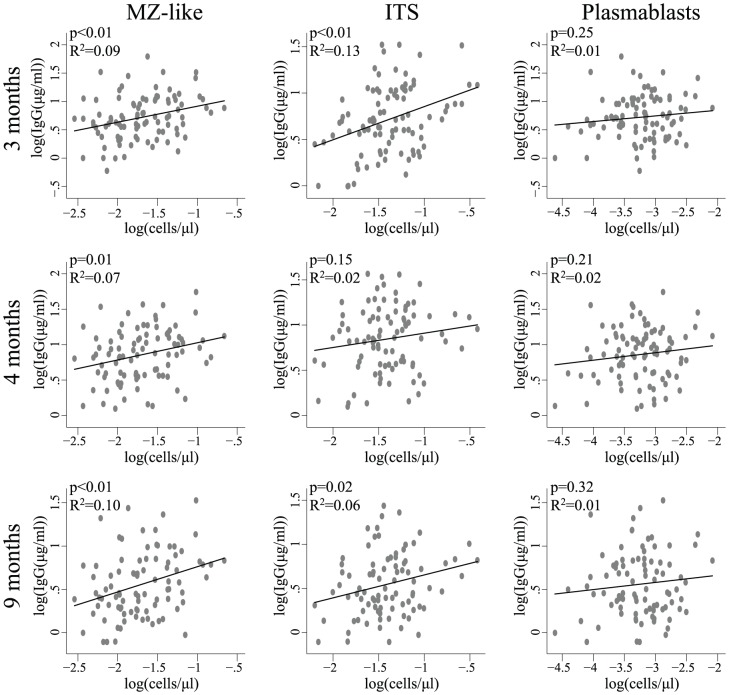
Linear regression plots: Pre-vaccination baseline B-cell subpopulations as predictors for IgG antibody concentration. Scatter plots with the best fitted line. Associations between the cell subset count (log cells/µl) plotted against the IgG vaccine-specific antibody concentration (log IgG(µg/ml)) at 3, 4, and 9 months. Two outliers have been removed from the analyses of the MZ-like and three from the isotype switched B cells.

The vaccine-specific IgG concentration measured at 3, 4, and 9 months post vaccination correlated positively with MZ-like B-cell count in unadjusted and adjusted linear regression analyses (unadjusted: p<0.05 in all analyses, Figure 2; adjusted: p = 0.01 in all analyses, Table 2). The percentage of MZ-like B cells were not associated with the vaccine-specific IgG concentration (p>0.05 in all analyses, Figure S2) and similarly was no association between the MZ-like B cells and the vaccine-specific OPA titer found (p>0.05 in all analyses, Figure S3)

The IgM-only B-cell count was not associated with vaccine-specific IgG concentration (p>0.05 in all analyses, Table 2).

Isotype-switched memory B cells demonstrated positive associations with the specific IgG concentration in unadjusted regression analyses at the 3 and 9-month follow-up visits (p = 0.01, Figure 2). In the adjusted analyses, isotype-switched B-cells predicted the specific IgG concentration at all follow-up visits (p<0.01 in all analyses, Table 2). The percentage of isotype-switched memory B cells was not associated with the specific IgG concentration in unadjusted regression analyses (Figure S2). In the adjusted analyses, however, we found a positive association between the percentage of isotype-switched memory B cells and the IgG concentration (p≤0.05, data not shown). Furthermore, the isotype-switched memory B-cell count was positively associated with the vaccine-specific OPA titer at the three-month follow up visit (p<0.01, Figure S3). We also found a positive correlation between the isotype-switched memory B-cell count and a minor reduction in IgG concentration from the 3-month visit to the 9-month visit (p<0.01, data not shown).

To further investigate whether the MZ-like or isotype-switched memory B cells were independent predictors of the vaccine response, both cell subsets were included in the multivariable model as predictors. In this analysis, the effect of the MZ-like cells was no longer present, whereas isotype-switched memory cells still predicted vaccine-specific IgG concentrations at the 3 and 9-month follow-up visits (p = 0.02 and p = 0.05, respectively). To determine whether the effect of isotype-switched memory cells was independent of the pre-vaccine CD4 T-cell count, this variable was added as a continuous parameter to the multivariable model. We found that isotype-switched memory B cells was a stronger predictor of the 3, 4, and 9-month IgG response than the pre-vaccine baseline CD4 T-cell count, inasmuch as only the isotype-switched memory cells was related to the IgG response (isotype-switched memory cells: p<0.02 in all analyses; CD4 T-cell count: p>0.05 in all analyses). Furthermore, in the unadjusted analysis, R^2^ of the isotype switched memory cells was marginally higher than or equal to R^2^ of the CD4 T-cell count at all follow-up visits (isotype switched: R^2^ = 0.08, 0.03, 0.07 respectively. CD4 T cells: R^2^ = 0.07, 0.03, 0.06). Thus, prevaccination isotype-switched memory-cell count is a CD4 T-cell-independent predictor of pneumococcal conjugate vaccine response.

### Vaccine responses were not associated with B cell activation

The transitional B cells did not predict the vaccine-specific IgG concentration at any of the three follow-up visits in unadjusted (data not shown) or adjusted analyses (Table 2). Similarly, no associations were observed between plasmablasts and the vaccine-specific IgG concentration at all three visits in unadjusted analyses (Figure 2). However, in adjusted analyses, plasmablast cell count was positively associated with the IgG response at all three visits (p<0.05 in all analyses, Table 2), whereas the percentages of plasmablasts showed no correlation to vaccine response (p>0.05 in all analyses, Figure S2). Neither did the plasmablast cell count show any association with the vaccine specific OPA titer (p>0.05 in all analyses, Figure S3). We also investigated whether the concentration of plasmablasts was an independent predictor of the IgG response by adding isotype-switched memory B cells as a predictor of vaccine response. In this analysis, the association between plasmablasts and antibody responses was no longer present, whereas isotype-switched memory B cells still predicted the vaccine response at 3 and 9 months (p = 0.01 in both analyses).

We observed no effect of CPG7909 on the association between B cell subsets and the specific IgG response, neither in the adjusted or unadjusted analyses (data not shown).

## Discussion

This study supports the essential roles of the B cell memory compartment and effective ART to achieve potent vaccine responses in persons infected with HIV. We found that isotype-switched memory B cells strongly correlated with a specific IgG response to PCV-7. MZ-like B cells and plasmablasts also predicted the response to PCV-7; however, this was not independent of isotype-switched memory cells. We observed higher numbers of isotype-switched memory B cells and MZ-like B cells in ART responders compared with impaired responders and ART-naïve patients. Finally, ART was associated with reduced B-cell turnover, demonstrated by lower levels of transitional B cells and plasmablasts.

HIV infection causes B-cell memory defects [12], and HIV-infected individuals had decreased antibody responses to T cell-independent antigens, such as the PPV-23 [19], [29]. Our study is the first to investigate the effect of B-cell subsets on the response to pneumococcal conjugate vaccination. The percentage of isotype-switched B cells was previously reported to be positively associated with the antibody response to another T-dependent vaccine, Tetanus toxoid, among HIV-infected patients [19]. Conversely, the response to PPV-23 did not depend on isotype switched but instead on MZ-like B cells [19]. We found that isotype switched and MZ-like B cells both predicted the IgG response to PCV-7. The isotype-switched B cells predicted the response independently of the CD4 T-cell count. We speculate that the association of the IgG response with both isotype-switched and MZ-like B cells reflects that T-dependent and independent mechanisms are participating in the mounting of a response to PCV-7. It has previously been reported that vaccination of HIV-infected patients with PCV-7 enhance the magnitude and breadth of specific IgG responses compared with PPV-23 alone [30]. Patients vaccinated with PCV-7 developed T-cell responses and an increased production of Th1 cytokines in response to the diphteria-derived carrier protein [30].

We found that patients responding to ART with an increase in CD4 cell count above 500 cells/µl had more isotype-switched and MZ-like B cells. These patients had a higher mean nadir CD4 T-cell count than impaired responders. The time since seroconversion was not known, but it is probable that part of the reason for the higher nadir CD4 T-cell count among responders was caused by earlier initiation of treatment than among impaired responders. Our study thus supports that treatment initiation at higher CD4 T-cell counts may be preferable to preserve B cell function. In the previously mentioned study comparing the effect of PPV-23 alone with PCV-7 among HIV-infected patients, a low nadir CD4 T-cell count predicted an impaired response in vitro to the carrier protein [30]. This is in accordance with the finding that patients treated with ART early during infection with HIV achieved stronger specific B-cell responses in vitro than patients treated during chronic infection [14]. Earlier treatment of HIV infection may prevent the irreversible damage to the memory B cell compartment and thereby increase the long-term humoral immunity of these patients [14].

Our results emphasize the importance of distinction between IgM-only memory cells and MZ-like B cells. ART-naïve patients had high numbers of IgM-only memory cells, whereas the MZ-like B cells did not differ between the treated and untreated patient groups. These findings indicate that the two cell types are affected differently by HIV infection. The number of MZ-like B cells was decreased in impaired responders compared with ART responders, and predicted the vaccine response. This is in contrast with the findings of Hart et al who did not report a difference in the percentage of MZ-like B cells according to CD4 T-cell count among ART-treated patients, however, in that study higher levels were found among untreated patients with high CD4 T-cell counts. Our results suggest a higher degree of immunological dysfunction in impaired responders compared with responders. The concept of a specialized “IgM memory subset” has been challenged by a series of studies [15], [17] and has recently been reviewed [16]. Consistent with the observed association between MZ-like B cells and the presence of IgG antibodies in our study, the MZ-like phenotype is proposed to be a circulating counterpart of the splenic MZ cells. IgM^+^ IgD^+^ CD27^+^ MZ-like B cells are not classical memory cells but represent an independent B-cell lineage, which develops through a germinal-centre-independent pathway [16]. This is in contrast to IgM-only memory cells, which are believed to develop through a germinal-center-dependent pathway but with a yet undefined role [16], [28]. MZ-like B cells play an important role in the response to encapsulated bacteria. Patients lacking MZ-like B cells are susceptible to severe pneumococcal infections [15]. This includes patients with common variable immunodeficiency and splenectomized patients. In both groups, a low or missing MZ-like B-cell count is associated with recurrent pneumonia and lack of anti-pneumococcal antibodies [15]. Additionally, children have low numbers of MZ-like B cells, and peptide conjugation of the pneumococcal vaccine substantially reduces the risk of systemic pneumococcal infection in children because the conjugate vaccine elicits a T-cell dependent response [15]. Even though PCV-7 is a conjugate vaccine, and MZ-like B cells primarily produce IgM, we found that the number of MZ-like B cells predicted the IgG response to PCV-7. This is, however, in accordance with the role of MZ-like B cells in facilitating the development of germinal centres.

B-cell turnover was reduced by ART, as evidenced by decreased concentrations of transitional B cells among treated patients. The concentration of plasmablasts was also lower among ART-treated patients. This is in accordance with the decrease in HIV-specific and non-specific antibody-secreting cells observed after ART initiation reported by Morris et al [31] and the finding of a decrease in the percentage of plasmablasts after initiation of ART reported by Moir et al [14]. It may seem conflicting with our other finding of a positive association between the concentration of plasmablasts and the vaccine specific IgG response in adjusted analyses. However, the analysis was adjusted for ART treatment and treatment response and thus we speculate that the positive association reflects that higher concentrations of plasmablasts within groups predict a higher potential for IgG production, which is needed for mounting a response to the subsequent vaccination.

The association between B cell subsets and PCV induced OPA responses was not as clear as it was for the quantitative vaccine-specific IgGs. Poor OPA responses have previously been shown to be associated with untreated HIV infection [8]. It has been suggested that HIV specifically interferes with the variable region gene family 3 which is involved in the synthesis of anti-pneumococcal immunoglobulins leading to dysfunctional antibodies with lower opsonophagocytic activity [32]. Thus, other mechanisms than pertubations of B cell subsets are likely to have an impact on OPA responses, which could explain why we did not find the same associations between B cell subsets for OPA as we did with IgG. IgG concentrations may be more relevant to study when correlations with B-cell subsets are assessed as immunoglobulins are the immediate products of terminally differentiated B cells, whereas OPA rely on several factors not controlled by B cells.

There were some limitations to our study. We analyzed the relationship between IgG response to PCV-7 and peripheral blood B cells since this is by far the most accessible compartment and the most widely studied. However, peripheral blood B-cell subsets are only proxies for the cells taking part in the response. Our data indicates that isotype-switched B cells is the only peripheral blood B-cell subset which can be used to predict the PCV-7 response in individual patients. We report significant associations to allow for further investigations in other studies. Another limitation was the small size of the group of impaired responders (n = 13), which limited the power of the analyses to detect differences between this subgroup and the two other groups. Nevertheless, we found several differences between this group and the treatment group.

The study did not include a healthy control group as the main study was designed to analyse the PCV-7 response among HIV patients. Healthy controls would however have made it possible to better isolate HIV-induced alterations in the distribution of B cell subsets. In the study we did not adjust for multiple comparisons. As our findings were often consistent between ART-receiving groups and between follow-up visits, we found it important to evaluate the general patterns of association between B-cell subsets and the vaccine response. Therefore we did not consider adjustment for multiple comparisons to be appropriate.

In conclusion, our study showed that isotype-switched memory B cells strongly predicted the PCV-7 vaccine response in HIV infected individuals and that the effect was independent of the CD4 T-cell count. MZ-like B cells and plasmablasts also correlated positively with the IgG response, although not independently of isotype-switched memory B cells. Patients who responded immunologically to ART (CD4 cell count >500 cells/µl) had increased numbers of both isotype-switched and MZ-like memory B cells compared with immunologically impaired responders (CD4 cell count <500 cells/µl after more than 3 years of continuous ART). ART was associated with reduced polyclonal B-cell turnover. Our findings emphasize that B-cell subset disturbances are associated with poor vaccine response among HIV-infected patients and support the idea that B-cell perturbations play an important and independent role in HIV pathogenesis.

## Supporting Information

Figure S1 **Flow cytometric gating hierarchy.** Gating showed for one representative individual on ART.
(EPS)

Figure S2 **Linear regression plots: Pre-vaccination baseline B-cell subpopulation percentages as predictors for IgG antibody concentration.** Scatter plots with the best fitted line. Associations between the cell subset percentage of the total B-cell population (log %) plotted against the IgG vaccine-specific antibody concentration (log IgG(µg/ml)) at 3, 4, and 9 months.
(EPS)

Figure S3 **Linear regression plots: Pre-vaccination baseline B-cell subpopulation as predictors for IgG antibody concentration.** Scatter plots with the best fitted line. Associations between the cell subsets (log cells/µl) plotted against the vaccine-specific opsonophagocytic activity (OPA) (log (OPA titer)) at 3, 4, and 9 months.
(EPS)

## References

[1] Sogaard OS, Lohse N, Gerstoft J, Kronborg G, Ostergaard L, et al (2008) Hospitalization for pneumonia among individuals with and without HIV infection, 1995–2007: a Danish population-based, nationwide cohort study. Clin Infect Dis 47: 1345–1353. 18834317 10.1086/592692

[2] Kohli R, Lo Y, Homel P, Flanigan TP, Gardner LI, et al (2006) Bacterial pneumonia, HIV therapy, and disease progression among HIV-infected women in the HIV epidemiologic research (HER) study. Clin Infect Dis 43: 90–98. 16758423 10.1086/504871

[3] Nuorti JP, Butler JC, Gelling L, Kool JL, Reingold AL, et al (2000) Epidemiologic relation between HIV and invasive pneumococcal disease in San Francisco County, California. Ann Intern Med 132: 182–190. 10651598 10.7326/0003-4819-132-3-200002010-00003

[4] Kaplan JE, Benson C, Holmes KH, Brooks JT, Pau A, et al (2009) Guidelines for prevention and treatment of opportunistic infections in HIV-infected adults and adolescents: recommendations from CDC, the National Institutes of Health, and the HIV Medicine Association of the Infectious Diseases Society of America. MMWR Recomm Rep 58: 1–207; quiz CE201–204. 19357635

[5] Barry PM, Zetola N, Keruly JC, Moore RD, Gebo KA, et al (2006) Invasive pneumococcal disease in a cohort of HIV-infected adults: incidence and risk factors, 1990–2003. AIDS 20: 437–444. 16439878 10.1097/01.aids.0000206507.54901.84

[6] Dworkin MS, Ward JW, Hanson DL, Jones JL, Kaplan JE (2001) Pneumococcal disease among human immunodeficiency virus-infected persons: incidence, risk factors, and impact of vaccination. Clin Infect Dis 32: 794–800. 11229848 10.1086/319218

[7] Falco V, Jordano Q, Cruz MJ, Len O, Ribera E, et al (2006) Serological response to pneumococcal vaccination in HAART-treated HIV-infected patients: one year follow-up study. Vaccine 24: 2567–2574. 16423429 10.1016/j.vaccine.2005.12.021

[8] Sogaard OS, Schonheyder HC, Bukh AR, Harboe ZB, Rasmussen TA, et al (2010) Pneumococcal conjugate vaccination in persons with HIV: the effect of highly active antiretroviral therapy. AIDS 24: 1315–1322. 20559037 10.1097/QAD.0b013e328339fe0b

[9] Crum-Cianflone NF, Huppler Hullsiek K, Roediger M, Ganesan A, Patel S, et al (2010) A randomized clinical trial comparing revaccination with pneumococcal conjugate vaccine to polysaccharide vaccine among HIV-infected adults. J Infect Dis 202: 1114–1125. 20795819 10.1086/656147 PMC2932785

[10] Madhi SA, Klugman KP, Kuwanda L, Cutland C, Kayhty H, et al (2009) Quantitative and qualitative anamnestic immune responses to pneumococcal conjugate vaccine in HIV-infected and HIV-uninfected children 5 years after vaccination. J Infect Dis 199: 1168–1176. 19265481 10.1086/597388

[11] Malley R (2010) Antibody and cell-mediated immunity to Streptococcus pneumoniae: implications for vaccine development. J Mol Med 88: 135–142. 20049411 10.1007/s00109-009-0579-4

[12] Moir S, Fauci AS (2009) B cells in HIV infection and disease. Nat Rev Immunol 9: 235–245. 19319142 10.1038/nri2524 PMC2779527

[13] Shen X, Tomaras GD (2010) Alterations of the B-Cell Response by HIV-1 Replication. Curr HIV/AIDS Rep 10.1007/s11904-010-0064-2 PMC3638746 21161615

[14] Moir S, Buckner CM, Ho J, Wang W, Chen J, et al (2010) B cells in early and chronic HIV infection: evidence for preservation of immune function associated with early initiation of antiretroviral therapy. Blood 116: 5571–5579. 20837780 10.1182/blood-2010-05-285528 PMC3031405

[15] Kruetzmann S, Rosado MM, Weber H, Germing U, Tournilhac O, et al (2003) Human immunoglobulin M memory B cells controlling Streptococcus pneumoniae infections are generated in the spleen. J Exp Med 197: 939–945. 12682112 10.1084/jem.20022020 PMC2193885

[16] Weill JC, Weller S, Reynaud CA (2009) Human marginal zone B cells. Annu Rev Immunol 27: 267–285. 19302041 10.1146/annurev.immunol.021908.132607

[17] Weller S, Braun MC, Tan BK, Rosenwald A, Cordier C, et al (2004) Human blood IgM “memory” B cells are circulating splenic marginal zone B cells harboring a prediversified immunoglobulin repertoire. Blood 104: 3647–3654. 15191950 10.1182/blood-2004-01-0346 PMC2590648

[18] Fournier AM, Baillat V, Alix-Panabieres C, Fondere JM, Merle C, et al (2002) Dynamics of spontaneous HIV-1 specific and non-specific B-cell responses in patients receiving antiretroviral therapy. AIDS 16: 1755–1760. 12218386 10.1097/00002030-200209060-00007

[19] Hart M, Steel A, Clark SA, Moyle G, Nelson M, et al (2007) Loss of discrete memory B cell subsets is associated with impaired immunization responses in HIV-1 infection and may be a risk factor for invasive pneumococcal disease. J Immunol 178: 8212–8220. 17548660 10.4049/jimmunol.178.12.8212

[20] Chong Y, Ikematsu H, Kikuchi K, Yamamoto M, Murata M, et al (2004) Selective CD27+ (memory) B cell reduction and characteristic B cell alteration in drug-naive and HAART-treated HIV type 1-infected patients. AIDS Res Hum Retroviruses 20: 219–226. 15018710 10.1089/088922204773004941

[21] Moir S, Malaspina A, Ho J, Wang W, Dipoto AC, et al (2008) Normalization of B cell counts and subpopulations after antiretroviral therapy in chronic HIV disease. J Infect Dis 197: 572–579. 18240953 10.1086/526789

[22] French N, Gordon SB, Mwalukomo T, White SA, Mwafulirwa G, et al (2010) A trial of a 7-valent pneumococcal conjugate vaccine in HIV-infected adults. N Engl J Med 362: 812–822. 20200385 10.1056/NEJMoa0903029 PMC2873559

[23] Sogaard OS, Lohse N, Harboe ZB, Offersen R, Bukh AR, et al (2010) Improving the immunogenicity of pneumococcal conjugate vaccine in HIV-infected adults with a toll-like receptor 9 agonist adjuvant: a randomized, controlled trial. Clin Infect Dis 51: 42–50. 20504165 10.1086/653112

[24] Konradsen HB, Sorensen UB, Henrichsen J (1993) A modified enzyme-linked immunosorbent assay for measuring type-specific anti-pneumococcal capsular polysaccharide antibodies. J Immunol Methods 164: 13–20. 8360502 10.1016/0022-1759(93)90270-h

[25] Skovsted IC, Kerrn MB, Sonne-Hansen J, Sauer LE, Nielsen AK, et al (2007) Purification and structure characterization of the active component in the pneumococcal 22F polysaccharide capsule used for adsorption in pneumococcal enzyme-linked immunosorbent assays. Vaccine 25: 6490–6500. 17655983 10.1016/j.vaccine.2007.06.034

[26] Martinez JE, Clutterbuck EA, Li H, Romero-Steiner S, Carlone GM (2006) Evaluation of multiplex flow cytometric opsonophagocytic assays for determination of functional anticapsular antibodies to Streptococcus pneumoniae. Clin Vaccine Immunol 13: 459–466. 16603613 10.1128/CVI.13.4.459-466.2006 PMC1459634

[27] Warnatz K, Schlesier M (2008) Flowcytometric phenotyping of common variable immunodeficiency. Cytometry B Clin Cytom 74: 261–271. 18561200 10.1002/cyto.b.20432

[28] Sanz I, Wei C, Lee FE, Anolik J (2008) Phenotypic and functional heterogeneity of human memory B cells. Semin Immunol 20: 67–82. 18258454 10.1016/j.smim.2007.12.006 PMC2440717

[29] Titanji K, De Milito A, Cagigi A, Thorstensson R, Grutzmeier S, et al (2006) Loss of memory B cells impairs maintenance of long-term serologic memory during HIV-1 infection. Blood 108: 1580–1587. 16645169 10.1182/blood-2005-11-013383

[30] Rabian C, Tschope I, Lesprit P, Katlama C, Molina JM, et al (2010) Cellular CD4 T cell responses to the diphtheria-derived carrier protein of conjugated pneumococcal vaccine and antibody response to pneumococcal vaccination in HIV-infected adults. Clin Infect Dis 50: 1174–1183. 20210645 10.1086/651418

[31] Morris L, Binley JM, Clas BA, Bonhoeffer S, Astill TP, et al (1998) HIV-1 antigen-specific and -nonspecific B cell responses are sensitive to combination antiretroviral therapy. J Exp Med 188: 233–245. 9670036 10.1084/jem.188.2.233 PMC2212446

[32] Subramaniam KS, Segal R, Lyles RH, Rodriguez-Barradas MC, Pirofski LA (2003) Qualitative change in antibody responses of human immunodeficiency virus-infected individuals to pneumococcal capsular polysaccharide vaccination associated with highly active antiretroviral therapy. J Infect Dis 187: 758–768. 12599049 10.1086/368331

